# Editorial Department

**Published:** 1846-06-01

**Authors:** 


### EDITORIAL DEPARTMENT.

A few remarks, by way of introduction, may not be inappropriate to the occasion of issuing the first number of our enlarged journal. The undertaking was commenced a year ago as an experiment. The result of a year's trial is a confirmation of the views which originated it. These views, as stated in the introductory to the first volume, were not peculiar to ourselves, but entertained in common with others to whose opinions we should be disposed to differ, were they not in accordance with our own, and to whose interest and co-operation the work is in a great measure indebted for the support which it has received. It should be added, that our associates in the enterprise are not less impressed than ourselves with the encouragement which exists for the continuance of the journal, and will not abate their valuable assistance in promoting its farther success.

The propriety of entering upon a second volume being apparent, it was equally clear that the work should be enlarged. The first volume afforded insufficient space for a satisfactory fulfilment of the functions of a journal, and, hence, if advisable that one be sustained here, it should obviously have larger dimensions. We have, accordingly, trebled its original size, and, also, engaged for it an improved typography.

Such are the circumstances under which we offer our editorial services for the continued, and, as we hope, increased patronage and favor of the medical public. We would here repeat, that if expectations of pecuniary compensation enter at all into our motives, they are certainly not proximate : not that we affect to be independent of, or indifferent to such considerations, nor that our humility is so extreme that we profess to hold our labors enterely beneath pecuniary reward ; but we wish it to be understood, that we are too much interested in the character of the journal to avail ourselves at present of any revenue which might be derived from it, except for the continued enlargement or improvement of the work. We desire this consideration to have its influence in inducing those who are, or may become its patrons, to endeavor to extend its circulation, and thereby enable us to make it more useful, and deserving of patronage.

Our aim, in short, is to establish the journal on a permanent foundation, and, if possible, to secure for it a position and influence which will in some measure not only satisfy our own wishes, but realize the expectations of its friends, who have taken a warm and active interest in its success. In pursuance of this aim, if life and health are spared, we intend to devote to it all the attention compatible with other and paramount duties, endeavoring, to the best of our ability, to render its pages interesting and instructive, relying with confidence on the indulgence of its patrons for the imperfections and faults necessarily incident to a limited experience in editorial duties, and to the disadvantages under which these duties are performed, when associated with those appertaining to medical practice, when necessity requires that the latter should always take precedence. Entertaining the conviction that to periodical literature, with its direct and indirect influences, medicine has been in the past, and it is to be expected will be for the future, especially indebted for its progressive advancement both as a science and profession, we would humbly hope to be instrumental in contributng somewhat toward medical progress, not as a rival candidate for favor, but as a coadjutor with those whose labors have been of longer duration than ours, and whose usefulness we can only hope to imitate, not excel.

In conclusion, we beg leave to quote the following from the introductory to our first volume, as expressive of the views and spirit by which we have, as we hope, thus far been governed, and which will continue to actuate us in our editorial capacity :—

" Our pages will be open to interchange of opinions and free discussion, not only of subjects strictly belonging to medical science, but of any involved in the progress or interests of the medical profession. Medical education, legislation, ethics, quackery, and the various projects of medical reform are among the subjects which furnish questions of interest and moment. We shall be desirous of appropriating to articles upon these topics as much space as our limits will allow, and to afford an opportunity for the expression of different views and opinions without restraint, except by the rules of decorum and propriety. We would add, that the journal is pledged to no interests apart from those which relate exclusively to the progress of medical science, and the advancement of the medical profession. It is not instituted for any sectional objects, or partizan views, but to serve as an organ for the impartial and untrammelled utterance of opinion on any matters pertaining directly or indirectly to its professed objects."

